# *In-situ* Quasi-Instantaneous e-beam Driven Catalyst-Free Formation Of Crystalline Aluminum Borate Nanowires

**DOI:** 10.1038/srep22524

**Published:** 2016-03-03

**Authors:** Ignacio G. Gonzalez-Martinez, Thomas Gemming, Rafael Mendes, Alicja Bachmatiuk, Viktor Bezugly, Jens Kunstmann, Jürgen Eckert, Gianaurelio Cuniberti, Mark H. Rümmeli

**Affiliations:** 1 IFW Dresden, Institute for Complex Materials, P.O. Box D-01171 Dresden, Germany; 2 Institute of Materials Science and Max Bergmann Center of Biomaterials, Dresden University of Technology, 01062 Dresden, Germany; 3 Centre of Polymer and Carbon Materials, Polish Academy of Sciences, M. Curie-Sklodowskiej 34, Zabrze 41-819, Poland; 4 College of Physics, Optoelectronics and Energy & Collaborative Innovation Center of Suzhou Nano Science and Technology, Soochow University, Suzhou 215006, China; 5 Theoretical Chemistry, Department of Chemistry and Food Chemistry, Dresden University of Technology, 01062 Dresden, Germany; 6 Erich Schmid Institute of Materials Science, Austrian Academy of Sciences (ÖAW) and Department Materials Physics, Montanuniversität Leoben Jahnstraße 12, A-8700 Leoben, Austria

## Abstract

The catalyst-assisted nucleation and growth mechanisms for many kinds of nanowires and nanotubes are pretty well understood. At times, though, 1D nanostructures form without a catalyst and the argued growth modes have inconsistencies. One such example is the catalyst-free growth of aluminium borate nanowires. Here we develop an *in-situ* catalyst-free room temperature growth route for aluminium nanowires using the electron beam in a transmission electron microscope. We provide strong experimental evidence that supports a formation process that can be viewed as a phase transition in which the generation of free-volume induced by the electron beam irradiation enhances the atomic mobility within the precursor material. The enhanced atomic mobility and specific features of the crystal structure of Al_5_BO_9_ drive the atomic rearrangement that results in the large scale formation of highly crystalline aluminium borate nanowires. The whole formation process can be completed within fractions of a second. Our developed growth mechanism might also be extended to describe the catalyst-free formation of other nanowires.

Aluminium borate nanowires (NWs) have a set of remarkable physical properties including high hardness, chemical inertness, high temperature stability and a low thermal expansion coefficient^1^,^2^,^3^. They are often used as reinforcement components in composite materials. Aluminium borate NWs have at least two different crystalline phases: Al_4_B_2_O_9_ and Al_5_BO_9_ of which both are usually produced simultaneously during synthesis. The latter is more thermodynamically stable and hence for this reason with high temperature synthesis more Al_5_BO_9_ NWs than Al_4_B_2_O_9_ NWs are formed.

There are a wide variety of synthesis approaches reported for the production of aluminium borate NWs. Aluminium borate NWs have been produced via glass crystallization^4^, calcination of powders^5^,^6^,^7^, porous compacts^8^,^9^ and sol-gels^10^,^11^ or through chemical vapor deposition over sapphire substrates^12^,^13^ to name a few. Interestingly, the use of catalysts is avoided in almost all reports, thus, a growth mechanism other than the vapor-liquid-solid (VLS) mechanism must be considered. Moreover, no evidence for VLS (like metal nanoparticles at the tips of the nanowires) has been found even when metal catalysts have been added to the precursors^6^,^8^,^9^.

The proposed growth mechanisms vary. Often, the so-called self-catalytic growth process is argued. The mechanism requires an incongruent melting of the precursor; i.e. its components must have different melting points. Small droplets are created out of molten boron oxide due to its low melting point at around 450 °C. Aluminium then dissolves within the droplet and reacts with the boron oxide within the droplet finally precipitating as aluminium borate. The process continues until the precursor runs out of B_2_O_3_ and the persistent droplets evaporate. Sometimes this logic is “reversed”, and the argument then proposes a sea of liquid boron oxide in which small Al droplets are formed. The droplets act then as catalyst sites for the growth of the NWs. There are significant weaknesses to these hypotheses. For instance, the relative amounts of boron oxide and aluminium oxide are always comparable and neither is minute. It is difficult to imagine how individual droplets of any phase can form under such circumstances; instead the precursor should turn into a paste as the temperature rises. Molten B_2_O_3_ should be ubiquitous. Even if the Al melts afterwards we end up with something closer to a binary liquid rather than Al droplets within a sea of liquid B_2_O_3_.

Here we show strong experimental evidence that supports a universal formation process that can be viewed as a phase transition in which a sudden liberation of free-volume generated by an energy input (an electron beam in this case) results in the crystallization of nanowires within a precursor particle. This growth mechanism can also be extended to describe the formation of other nanowires, for example, WO_3_ nanowires produced inside a TEM^14^ as well as other synthesis routes^8^,^9^,^10^,^11^.

The technique we introduce here to produce the alumnuim borate NWs is in itself a breakthrough. We synthesized the NWs inside a transmission electron microscope (TEM) working at 300 kV using only the imaging electron beam to drive the synthesis reaction. This setup allowed us to observe the formation of the nanowires in real time, something never previously reported. Moreover, through an appropriate manipulation of the electron beam one can instigate further growth on some of the NWs.

## Results

By performing simple manipulations of the imaging electron beam one can nucleate and grow Al_5_BO_9_ NWs in a near instantaneous fashion from amorphous aluminium borate specimens inside a transmission electron microscope. When the Al_5_BO_9_ NWs are supported over the lacey C of the TEM grid their growth can be further extended.

**Figure 1:**
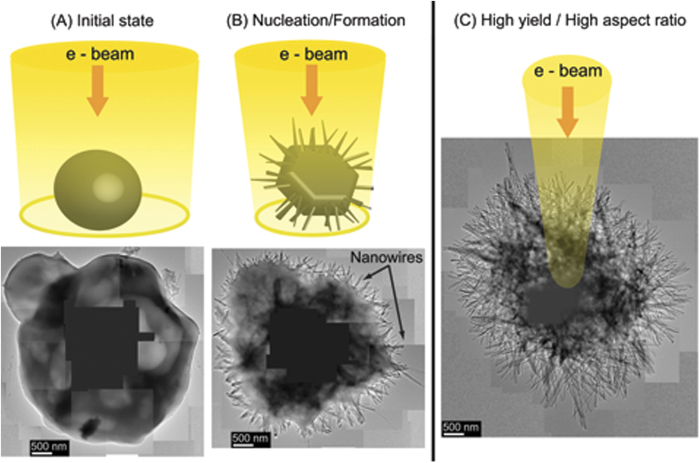
Single step formation. (**A**) The smooth precursor remains stable when the beam is spread out. (**B**) The precursor transforms near instantly as one diminishes the beam waist once the reaction current density threshold is reached. Typically, the transformation occurs when the diameter of the beam waist is slightly larger than that of the precursor specimen under investigation. After the reaction a large number of NWs stick out of the precursor pointing in all directions, in addition, there is a sizeable volume loss in the precursor. (**C**) The yield and aspect ratio of the NWs from a single precursor can be maximized by speeding up the reduction of the beam waist and turning the beam down to a nanometer probe. In these cases the details of the transformation happen too fast to be tracked by the naked eye.

Upon irradiation, the precursor material remains stable when the imaging electron beam is spread out, i.e. if the current density is below 6 × 10^−6 ^A/cm^2^ (See Fig. 1A) (the total current is obtained from TEM fluorescent screen readings). When the electron beam waist is gradually reduced, viz., the current density is increased; the precursor undergoes a radical transformation. A large number of NWs suddenly nucleate/grow such that large sections of the NWs protrude outward from the precursor forming an urchin like structure (See Fig. 1B). The nucleation can be triggered with current densities as low as 2 × 10^−4 ^A/cm^2^. However, the exact value of the current density varies from one precursor piece to another and it is never much higher than the threshold given above. The NWs form almost instantaneously if one increases the current density fast enough. Nevertheless, it is possible to glimpse the intermediate steps of the nucleation/growth process by gently decreasing the electron beam waist as can be seen in Movie S1 in which the current density increases at an approximate rate of 8 × 10^−6 ^A/cm^2^·s. Surpassing this current density growth rate generally results in the production of larger yields of NWs with a higher aspect ratio as for example seen in Fig. 1C.

**Figure 2:**
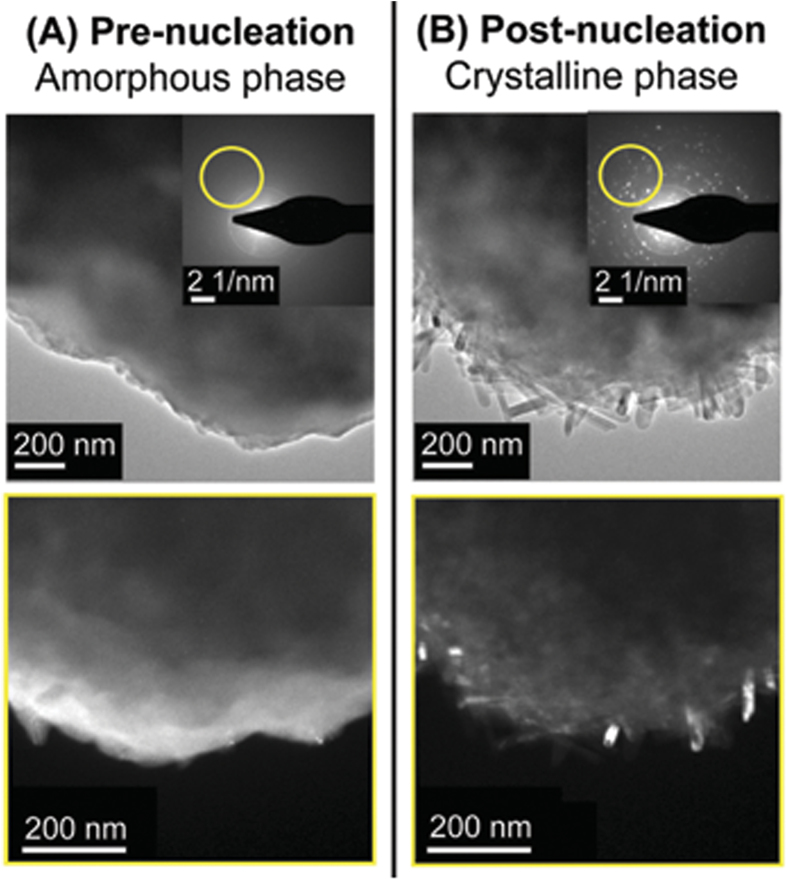
Amorphous to crystalline phase transition. (**A**) Prior to nucleation the precursor remains amorphous as can be seen from the featureless diffraction pattern in the inset. The region of the diffraction pattern encircled in yellow originates from the features highlighted in the image below (Selected Area Electron Diffraction, or SAED), providing a visual view of the amorphous character of the precursor. (**B**) A multitude of bright spots appear in the diffraction pattern after the NWs have been nucleated. The SAED study reveals crystalline NWs are responsible for the selected set of diffraction spots once the amorphous-crystalline phase transition has been completed.

The nucleation process can, in a broad sense, be described as a kind of phase transition. As already mentioned, the precursor is an amorphous material made out of O, Al and B atoms. The electron beam upon being squeezed, triggers and drives a great deal of microscopic and rapid restructuring, such that the O, Al and B atoms arrange themselves in a crystalline configuration forming highly crystalline aluminium borate NWs as confirmed by selected area electron diffraction studies before and after the electron beam treatment (beam squeeze) (e.g. Fig. 2). Some remnant amorphous material tends to remain and seems to form a scaffold that holds the NWs together in a single cluster that resembles an urchin.

**Figure 3:**
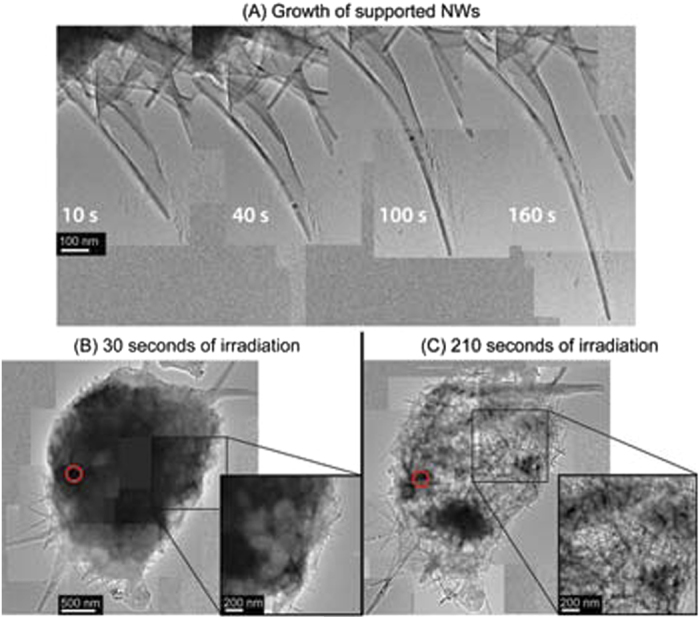
Extended growth of supported NW growth and progressive precursor thinning. (**A**) The length of the supported NWs shown grows visibly as the total condensed irradiation (red circle) increases. (**B**) After 30 seconds of condensed irradiation the precursor appears considerably compact. Nevertheless, areas of lighter contrast can already be seen near the edges. (**C**) After 210 seconds of condensed irradiation the precursor is extensively eroded indicating a substantial part of its material has been lost due to the action of the condensed beam. A dense matt of NWs extends all through the precursor forming an urchin-like structure.

After the quasi-instantaneous formation process is completed many of the NWs stick out of the urchin-like structure directly into the vacuum. Sometimes a few of the NWs happen to lie on a section of lacey C on the TEM grid. In this case continued electron beam exposure can further extend the growth of the NWs lying over lacey C (See Fig. 3A). To achieve the extended growth of the supported NWs the electron beam must be condensed to a small probe diameter of around 100 nm (~175 A/cm^2^) and placed over remnant amorphous material (e.g. nearer to the center of the urchin structure) and held there for a period of time (at least 10 s). The growth rate (in terms of NW length) of these supported NWs can be above 10 nm/s while free-standing NWs have significantly lower growth rates of around 1.5 nm/s and they tend to cease further growth rather quickly (see Fig. S1) . The supported NWs also tend to show some broadening (see Fig. S2). Simultaneously as there is a gradual thinning of the amorphous precursor discernible by the diameter reduction and lighter contrast of the amorphous precursor material in the urchin structure. The thinning is due to removal of amorphous precursor material by the electron beam. A complex network of entangled NWs is revealed as the amorphous precursor thins away (See Fig. 3B,C).

**Figure 4:**
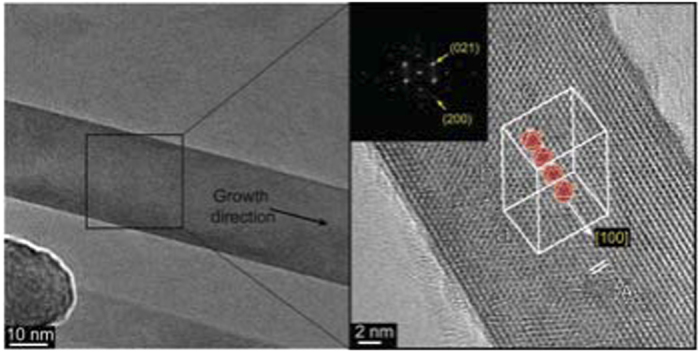
Crystalline NW structure and growth direction. The crystalline structure of the NW is clearly discernible in the high resolution TEM image on the right. The NW axis lies along the [100] direction as determined from the indexed FFT pattern in the top left corner (the [200] and [100] directions are parallel). A schematic drawing of the unit cell has been superimposed on the image to emphasize the parallelism between the NW’s growth direction and the octahedral chains that form its backbone.

The composition of the free-standing as well as of the extended growth NWs were investigated by electron energy loss spectroscopy (EELS) and Energy Filtered TEM imaging (EFTEM), see for example Figs S3 and S4 respectively. The spectroscopic studies confirmed that Al, B and O form the bulk of the NWs. In addition, detailed studies of the reconstructed fast Fourier transform (FFT) patterns from high resolution micrographs from numerous NWs were conducted to help identify their aluminum borate phase. From this analysis we can confidently establish that ca. 70% of the NWs are of the Al_5_BO_9_ phase. The remaining 30% could not be conclusively labeled as either Al_5_BO_9_ or Al_4_B_2_O_9_. Around 85% of the NWs that were identified with the Al_5_BO_9_ phase show growth along the longitudinal axis to be in the [100] crystallographic direction which is in agreement with most published data on Al_5_BO_9_ NWs. A typical example of an Al_5_BO_9_ NW from this work with its main axis along the [100] direction is presented in Fig. 4. A more detailed explanation on how the FFT analysis was carried out can be found in Fig. S5.

## Discussion

The key to understand the formation of aluminium borate NWs lies on their crystalline structure with respect to the NW axis. Both Al_4_B_2_O_9_ and Al_5_BO_9_ crystalline phases share an important characteristic, namely, they are built over a backbone of AlO_6_ octahedral units. The octahedral units are tightly packed along linear chains that run parallel to the *b* axis of the unit cell in the case of Al_4_B_2_O_9_ 15 and to the *a* axis in Al_5_BO_9_ 16 (see Fig. S6). Experimentally there is, for the most part, a correlation between the crystalline structure of the nanowires and their growth direction. This correlation is much clearer in the case of Al_5_BO_9_ NWs. Indeed, seven out of eight reports in the literature find the NWs grow along the [100] direction, i.e. parallel to the structure’s backbone. The situation of Al_4_B_2_O_9_ NWs is not as clear-cut, however, here we now focus our discussion on the Al_5_BO_9_ phase since it is the prevalent phase not only in our experiments but throughout the literature.

Despite the numerous variations in experimental approaches, the underlying rationale of the synthesis process of Al_5_BO_9_ NWs is similar in all cases, namely, one enhances the atomic mobility within bulk quantities of an either amorphous^2^,^4^,^10^,^11^ or crystalline^7^,^8^,^13^ precursor (powders, gels, pellets) by injecting energy into it. A considerable amount of free-volume is generated within the precursor during the process (through evaporation, for example). The mobile species have then more freedom to move, thus, facilitating the chemical reactions that ultimately produce NWs. In our approach the electron beam supplies the energy input onto the precursor rather than heat generated in a high temperature oven.

Our precursor can be described as a speck of material consisting of a homogeneous amorphous mix of Al, B and O with an approximate atomic ratio of 1:9:15 (4% of Al, 36% of B and 60% of O) with sizes ranging from less than a micron up to several microns across. Exposure to an electron beam with an appropriate current density triggers the generation of free-volume within the precursor. Experimentally we observed the free-volume generation as lighter contrast patches forming in the specimen under examination (Fig. S7). The free volume formation in the specimen can occur through electron stimulated desorption of material. One such pathway is the so-called Knotek-Feibelman (K-F) mechanism^17^ in which an electronic vacancy created in a boron atom leads to the emission of a cascade of Auger electrons from the valence band of the oxygen atom^18^. As a result, the oxygen atom can turn into a neutral atom that is easily desorbed or into a positive ion which is then repelled by the increasingly strong positive potential generated by electrostatic charging^19^ (the process is thoroughly explained in the supplementary information). In addition, ionized oxygen atoms can recombine to produce neutral O_2_ that is readily desorbed. The rate at to which the free volume is generated through these mechanisms can be accelerated by rapidly increasing the current density impinging on the specimen, in other words, by rapidly squeezing the electron beam. Moreover, while exposing the specimen to an electron beam, the mobility of atoms within the specimen is enhanced. This can occur by direct transfer of kinetic energy through nucleus-electron collisions (particularly effective in the case of boron atoms^20^) or by producing mobile reactive species through ionization. In addition, the development of a strong positive electrostatic potential can lead to atom ejection as the system seeks to obtain electrostatic equilibrium. The newly formed free volume and active species with increased atomic mobility provide the conditions for the formation of aluminum borate NWs as the irradiated specimen seeks to form a stable configuration through atomic rearrangement. Crystalline phases have lower internal energy as compared to amorphous configurations and therefore if the relative amount of elements approaches the stoichiometry of a stable phase, the likelihood of the restructuration process ending in the formation of a crystalline phase is high. In fact, this kind of athermal crystallization driven by the action of an electron beam has been extensively documented^20^,^21^,^22^,^23^,^24^. Electron beams can also lead to limited heating. To check this we conducted temperature calculations from the electron beam sample interaction and they show that the current densities used here do not raise the precursor temperature by more than 37 K even at the high current density of ~177 A/cm^2^ (see supplementary info) and so we can discard heating as playing any significant role in the NW formation.

We now discuss the irradiation-triggered crystallization process for the formation of Al_5_BO_9_ NWs in more detail. Initially, upon electron beam irradiation small structural gaps within the precursor form generating small semi-isolated areas (free-volume) due to electron beam stimulated desorption. Some atoms/ions within these regions are mobile and are reactive as a consequence of knock-on displacement and ionization events. The atoms in each semi-isolated region or islet can rearrange into a more stable structure^24^. Fast oxygen desorption and also of B but to a lesser extent (similar to what has been observed in boron oxide-based materials exposed to electron beams)^19^,^25^ serve to locally bring the initial atomic Al:B:O ratio (1:9:15) closer to 5:1:9 which is the most thermodynamically stable. In regions where the 5:1:9 stoichiometric relation is approached the atomic rearrangement will tend toward the thermodynamically stable crystalline configuration of Al_5_BO_9_. A similar atomic restructuration argument can be used to explain the growth of Ag filaments induced by exposing *α*-Ag_2_WO_4_ to electron beam irradiation. In that work, the electron beam-induced extrusion of Ag changes the relative amounts of Ag, W and O in the irradiated crystal leading to an amorphous structure since the compound departs from a stoichiometric relation that favors a stable crystalline phase^26^.

**Figure 5:**
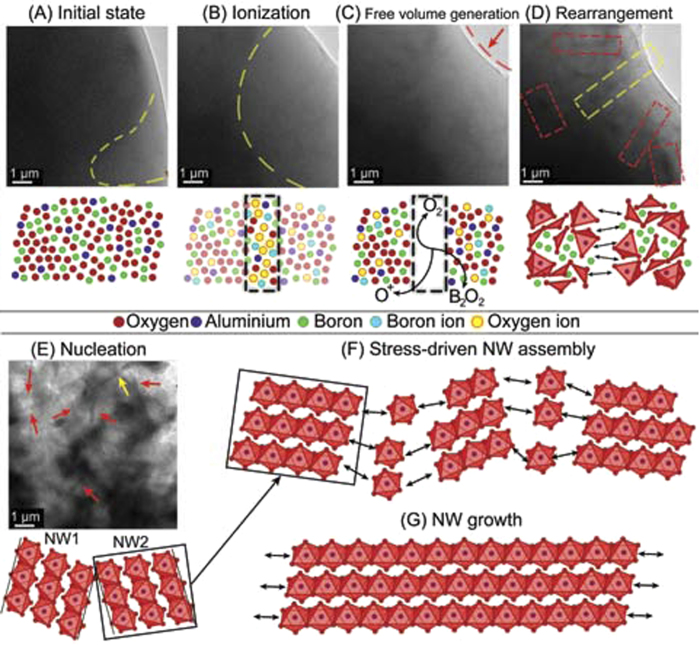
The NW formation process. The formation process of a group of NWs is illustrated in a sequence of screenshots taken from movie S1. (**A**) *Initial state*. The precursor is stable an amorphous when the beam is well spread. The dashed yellow line separates a region of dark contrast (left) from a thinner section of lighter contrast (right). (**B**) *Ionization*. As the current density increases there is a larger amount of atoms being ionized and displaced due to interactions with the impinging electrons. Inonized atoms are more reactive and mobile setting the stage for atomic rearrangement. A small degree of material desorption is stimulated by the beam (thinner region of lighter contrast extends). (**C**) *Free volume generation*. The newly created radicals react within the precursor. Larger amounts of species that can escape the specimen such as O^+^, O_2_ and B_2_O_2_ are generated. Note how the curvature of the precursor’s edge is further indented indicating a considerable degree of volume loss. (**D**) *Rearrangement*. Small regions of the specimen start to crystallize as the specimens atoms rearrange into a more stable configuration. Those regions have a slightly darker contrast (areas inside the dashed line boxes) indicating that the atoms have acquired a more closed-packed configuration. The elemental blocks that form the crystalline structure of the Al_5_BO_9_ NWs start to assemble in different islets. (**E**) *Nucleation*. The octahedral chains that form the backbone (and the groups that interlink them) are fully assembled in those regions that previously appeared as dim shadows. The shadows have now turned into recognizable NWs as the backbone chains grow (The NW pointed at by the yellow arrow formed from the shadow in the yellow dashed-line box). (**F**) Short pieces of backbone are assembled and stacked in various “islets”. There are “attractive” forces at the ends of the chains due to the close-packed structure of the octahedral groups. More and more octahedral groups and backbone sections are brought together. (**G**) A long NW results from the alignment and bridging of multiple shorter backbone sections.

In our study, once the crystallization is set in motion, AlO_6_ octahedra come together to assemble linear chains that form a backbone. This same process occurs for all the islets and leads to a preferential alignment of the forming backbone chains of neighboring islets as they “pull” elements to assemble their own backbone. This “pulling” effect can broadly be referred to as a “stress” that arises due to the inherent asymmetry introduced by the closely packed architecture of the AlO_6_ octahedral units that make up the backbone chains characteristic of the crystalline structure of Al_5_BO_9_. Consequently, atoms are added along the backbone direction at a faster rate than they are added transversally to it. Neighboring islets will come into contact as they crystallize; if they are nearly collinear then the short backbone sections can join at their extremes to fuse into a single NW extending along the [100] direction. This whole process is summarized in Fig. 5.

Two factors help to explain why transversal crystallization (growth) is less effective than the longitudinal crystallization. The first has to do with structural defects. The term “defect” is used to encompass a large variety of irregularities that interrupt the smoothness of a structure such as: cracks, pores, grain boundaries, impurities and even an inhomogeneity on the elemental distributions of Al, B and O. Moreover, such structural features are to be expected in all the precursors discussed in the literature. In our case most of the defects are small gaps or an inhomogeneous elemental distribution within the precursor. The “pulling” forces along the backbone chains of the NWs main axis are strong enough so as to bridge the gaps between neighboring crystallites joining them together. However, this is not the case along the direction transversal to the backbone chains since the larger distance between parallel chains might prevent them from coming together if structural defects are found in between them. The second factor has to do with the fact that the nucleation happens ubiquitously throughout the precursor, and, that the relative orientations between the main axes of different NWs vary stochastically. One can imagine that two neighboring islets start to crystallize but their backbones are significantly misaligned. By the time the transversal crystallization of both islets has extended enough so that the crystallites come into direct contact the twist angle will frustrate their fusion, instead, they preserve their individuality.

The rate of free volume generation will have important effects on the efficiency of the formation process discussed above. If the rate is too low then the islets fail to form effectively. The crystallization process is still triggered but the amount and extent of the defects is not large enough so as to impede the transversal fusion of neighboring crystallites. The end result will be broad whiskers (much as those often found in the literature) instead of NWs with high aspect ratios. Thermal synthesis favors the formation of such whiskers since the energy input ramps up more gentle as compared to the relatively rapid energy input delivered by an electron beam.

Our growth model also explains how the addition of impurities reduces the mean diameter of NWs fabricated in conventional thermal routes. This occurs because the presence of impurities diminishes the average size of islets containing only Al, B and O. This effect has been repeatedly observed when introducing metallic elements in the precursor^6^,^8^,^9^.

The extended growth after the initial quasi-instantaneous NW formation that is triggered by irradiating remnant amorphous material with a condensed electron beam relies on electrostatic breakdown due to charging effects. In short, the beam triggers a cascade of events that culminates on the migration of Al, B and O to the tips of the NWs rough electrostatic repulsion causing them to grow. The phenomenon can be dissected into two parts: the generation of feedstock material (Al, B and O) and its transport. A somewhat intricate interplay between radiolysis and specimen charging can be used to explain both issues in a cohesive manner. Radiolysis through the K-F mechanism explains the production of oxygen, boron and aluminium ions that serve as feedstock material for the growth of the NWs. The radial transport of this ionic feedstock material is driven by an increasingly strong electric field emanating from the irradiated spot as a consequence of specimen charging^19^. A secondary force that moves the newly generated ions in the radial direction is provided by their chemical gradient profiles which peaks at the irradiated volume (these points are examined more thoroughly in the supplementary information). The constant outflow of ions (mainly O) from the amorphous remnant precursor explains its thinning down with continued electron irradiation. One expects more reactivity near the edges of the precursor since repulsive forces decay with distance where ionic species will have lost a good deal of their kinetic energy.

We now explain why supported NWs grow at a much faster rate than their free-standing counterparts. Upon irradiation the produced ionic species are able to migrate superficially along all the NWs (both freestanding and supported). For the supported NWs the carbon film support facilitates surface transport by providing a larger surface along which the ions can move. The aluminium ions benefit most from this increased supply surface because there is much less Al available as compared to B and O. Moreover, the C film can adsorb B and O species, thus, holding them in place until sufficient quantities of Al can reach reaction sites where B and O are adsorbed (mainly at the NWs tips). This Al enhancement is not available to the free-standing NWs hence their extended growth is minute because they neither benefit from the multiplicity of transport channels made available by the large area of the support, nor is there is anything that prevents the fast vaporization of B and O species that react to produce volatile B_2_O_2_ species before enough Al ions reach the NWs to promote the formation of Al_5_BO_9_.

## Conclusion

In this work we show the room temperature quasi-instantaneous *in situ* catalyst-free formation of highly crystalline aluminium borate nanowires from amorphous precursors upon electron beam irradiation. The remarkable data allow us to develop a growth mechanism that can be universally applied to other growth approaches for aluminum borate nanowires and probably beyond just aluminum borate NWs, for example, the catalyst-free growth of WO_3_ NWs. In this work, we use an electron beam to drive the reaction. The developed formation mechanism can be described as a large scale amorphous-crystalline phase transition that takes place almost instantaneously at room temperature. The process is driven by the internal forces that arise as the atoms in the precursor rearrange themselves in order to lower the internal energy of the system after it has been structurally disrupted by the electron beam. The ubiquitous presence of internal forces is unavoidable due to the closed-packed nature of the octahedral chains that form the backbone of the crystalline structure of the Al_5_BO_9_ phase. In addition, after the quasi-instantaneous formation of the NWs, one can achieve extended growth of NWs supported over the lacey C of the TEM grid by using a highly condensed electron beam on the remaining precursor material. Growth occurs predominantly through surface diffusion of material of positively charged feedstock material. This fundamental *in-situ* study provides new and important insight for the formation of NWs where common growth modes such as VLS growth break down. Thus, this work provides new and important understanding for the catalyst-free growth of crystalline nanowires.

## Methods

600 mg pellets were made out of 90% B_2_O_3_ and 10% Al_2_O_3_ (atomic percentages) by compressing them at room temperature in a die under 2 tons of pressure. The pellets were laser-ablated in a setup described elsewhere^25^. The ablation process lasted for around 4 minutes. The material collected in the cold finger was a fine whitish gray powder that constitutes an amorphous precursor we use to produce Al_5_BO_9_ NWs. The precursor was put in a standard TEM Cu/lacey carbon grid and then loaded into an FEI-Tecnai F30 Transmission Electron Microscope operated at 300 kV. The NWs synthesis process was carried out at room temperature and inside the TEM column while being exposed to the imaging electron beam.

## Additional Information

**How to cite this article**: Gonzalez-Martinez, I. G. *et al*. *In-situ* Quasi-Instantaneous e-beam Driven Catalyst-Free Formation Of Crystalline Aluminum Borate Nanowires. *Sci. Rep*. **6**, 22524; doi: 10.1038/srep22524 (2016).

## Supplementary Material

Supplementary Information

Supplementary Movie S1
