# Supplementary material for: In-situ Quasi-Instantaneous e-beam Driven Catalyst-Free Formation Of Crystalline Aluminum Borate Nanowires
Source: Sci Rep. 2016 Mar 3;6:22524. doi: 10.1038/srep22524 (PMC4776144; doi:10.1038/srep22524)
Supplement: Supplementary Information [file srep22524-s1.doc]

**SUPPLEMENTARY INFORMATION.**

**In-situ quasi-instantaneous e-beam driven catalyst-free formation of crystalline Aluminum-borate nanowires.**

*Ignacio G. Gonzalez-Martinez,^1, 2^ Thomas Gemming,^1^  Rafael Mendes,^1^ Alicja Bachmatiuk,^1, 3, 4^ Viktor Bezugly,^2^ Jens Kunstmann,^2, 5^ Jürgen Eckert,^1, 2^ Gianaurelio Cuniberti,^2^ and Mark H. Rümmeli.^4,1,3^**

^1^ IFW Dresden, Institute for Complex Materials, P.O. Box D-01171 Dresden, Germany.

^2^ Institute of Materials Science and Max Bergmann Center of Biomaterials, Dresden University of Technology, 01062 Dresden, Germany.

^3^ Centre of Polymer and Carbon Materials, Polish Academy of Sciences, M. Curie-Sklodowskiej 34, Zabrze 41-819, Poland.

^4^ College of Physics, Optoelectronics and Energy & Collaborative Innovation Center of Suzhou Nano Science and Technology, Soochow University, Suzhou 215006, China.

^5^ Theoretical Chemistry, Department of Chemistry and Food Chemistry, Dresden University of Technology, 01062 Dresden, Germany.

**Corresponding Author**

*Correspondence and requests for materials should be addressed to M.H. Rümmeli (mhr1@suda.edu.cn).

**Movie S1.** The movie captures the early formation stages of the nanowires. The nanowires are observed to slowly nucleate as the beam waist is slowly narrowed down, i.e. the current density increases at a rate of approximately 8 x 10^-6^ A/cm^2^·s. The movie was recorded at a frame rate of 2 frames per second.

The presence of the lacey C support of the TEM grid greatly facilitates the transport of feedstock material by increasing the area along which the feedstock material can be transported when extending the growth process using a condensed electron beam to irradiate the precursor ***after*** the initial quasi instantaneous growth of NWs with a broad electron beam. The increased transport pathways provided by the underlying support is reflected by the much larger NW length of supported NWs as compared to unsupported NWs - see figure S1.


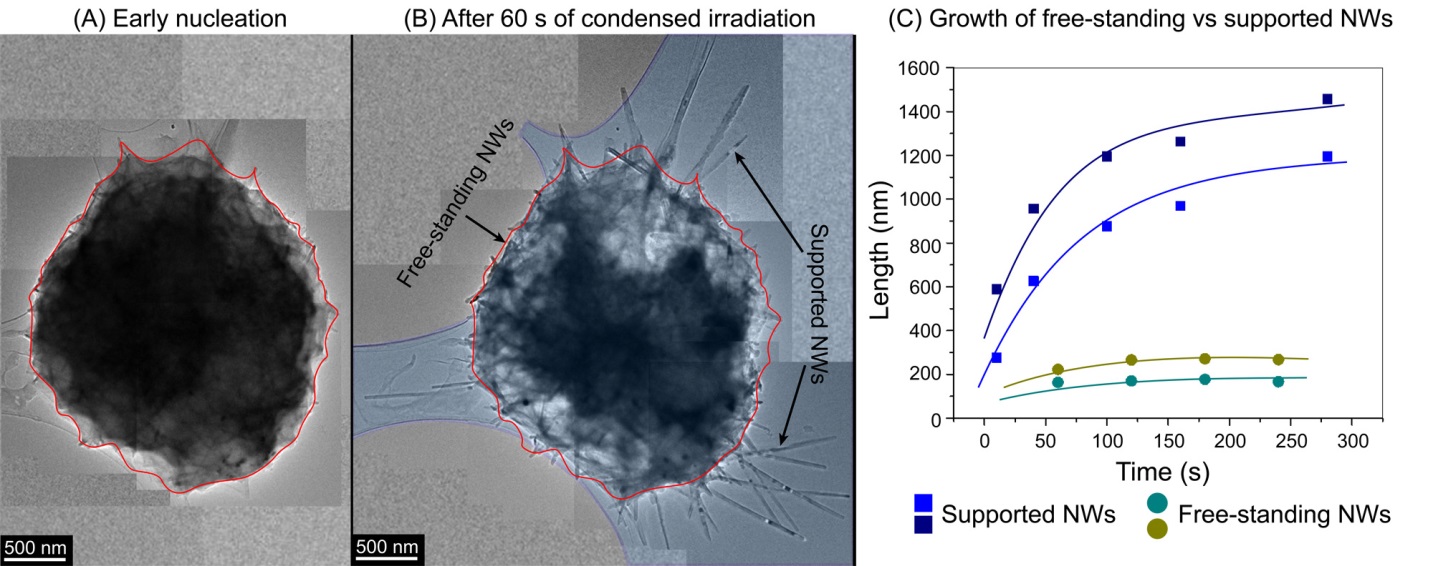


**Figure S1. Growth rate. (A)** The tips of some short NWs appear peering out from the edges of a precursor speck at an early state of nucleation. The red outline approximately delimits the maximum reach of the precursors contour including the tips of the protruding NWs. **(B)** After 60 seconds of condensed irradiation some NWs have grown way beyond the red outline that marked their post-nucleation length. The NWs that are supported over the lacey C (blue-shaded region) reach much longer CEOS third-order spherical aberration correctors for the objective lens (CETCOR) and the condenser system (CESCOR). The temperature in the microscope’s column was at room temperature.

Aside from increasing the NW length in the extend growth mode (condensed electron beam), most NWs show a degree of broadening which is typically between 5 - 15 nm. (see figure S2).


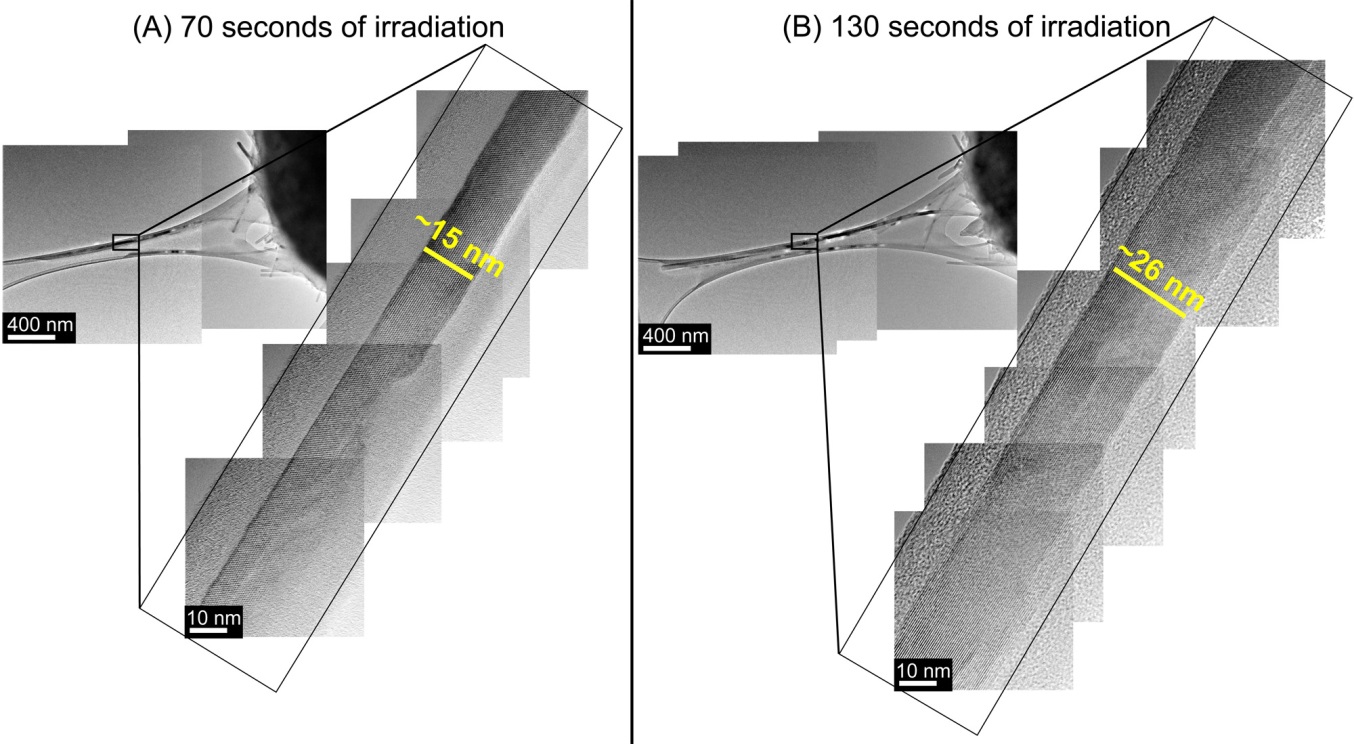


**Figure S2. Broadening of NWs.** **(A)** After 70 seconds of condensed beam irradiation a supported NW has a mean width of 15 nm. **(B)** After a further 60 seconds of condensed beam irradiation the width has increased by ca. 11 nm. Structural defects such as jagged surfaces smooth out with increasing irradiation time. This phenomenon can also be appreciated in the NW above.

The chemical composition of the NWs from both the initial quasi-instantaneous NW formation and the (subsequent) extended growth of NWs using a condensed electron beam as opposed to a broad beam were investigated using electron energy loss spectroscopy (EELS) as well as by obtaining elemental maps using Energy-Filtered TEM (EFTEM) techniques. The first part of these studies was carried out in free-standing NWs formed quasi-instantaneously with a broad irradiation beam. The analyses show that the NWs are made of Al, B and O e.g. figure S3.


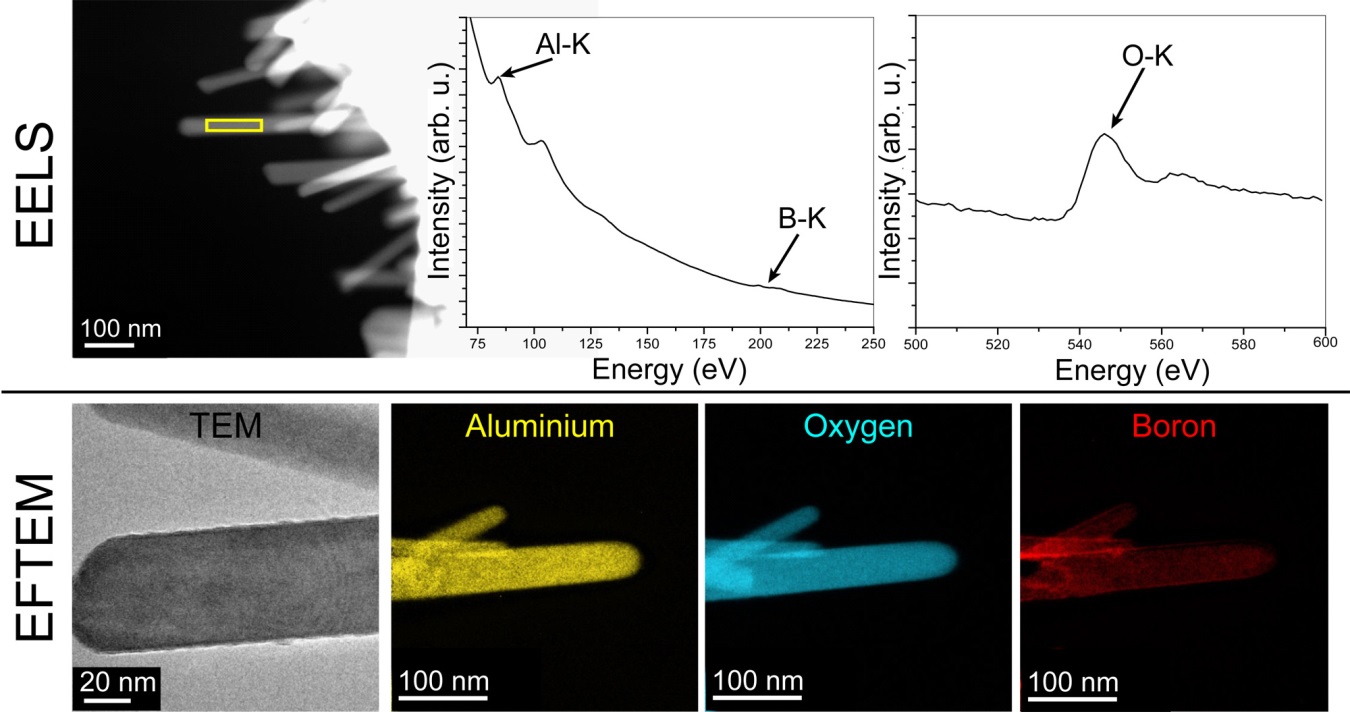


**Figure S3. Composition of the NWs**. The peaks corresponding to Al -K, B-K and O-K edges are observable in the EELS spectrum (top row). Al, B and O are uniformly distributed along the NWs as can be observed in the elemental maps produced by EFTEM analysis (bottom row).

For completeness, the EFTEM technique was used to study the composition of supported NWs that underwent extended growth when using a a condensed electron beam after the initial formation process. The study in figure S4 shows the chemical composition of the aluminum borate NWs remains uniform.


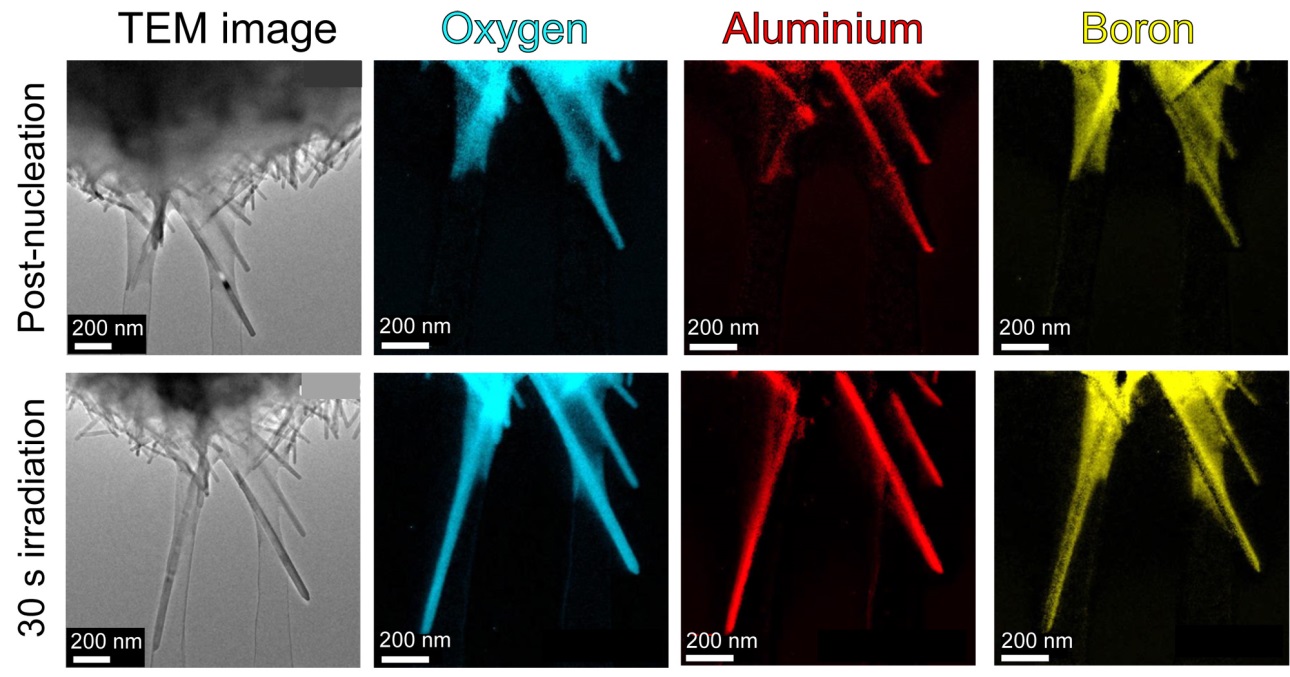


**Figure S4. EFTEM through growth.** A pair of extended NWs show significant growth as the condensed irradiation time increases. The elemental maps obtained through EFTEM show that significant amounts of B and O are profusely present over the lacey C as well as forming the NWs themselves. In contrast, Al is scarce on the support but uniformly distributed along the NWs.

**Identification of the aluminium borate phase.**

A careful analysis of the FFT patterns obtained from high resolution micrographs of the NWs allows one to identify their phase. The majority of them were identified as Al_5_BO_9_ NWs. The analysis consists of estimating the d-spacings and angles between crystalline planes. After the values for the spacings were measured they were comparted to various crystallographic data cards for Al_5_BO_9_ and Al_4_B_2_O_9_. A set of planes is selected based on their match with the estimated values for the d-spacings. Then the angles subtended between the selected planes are computed. The correct crystallographic phase is determined from the comparison of both the above criteria with the FFT patterns (see figure S5).


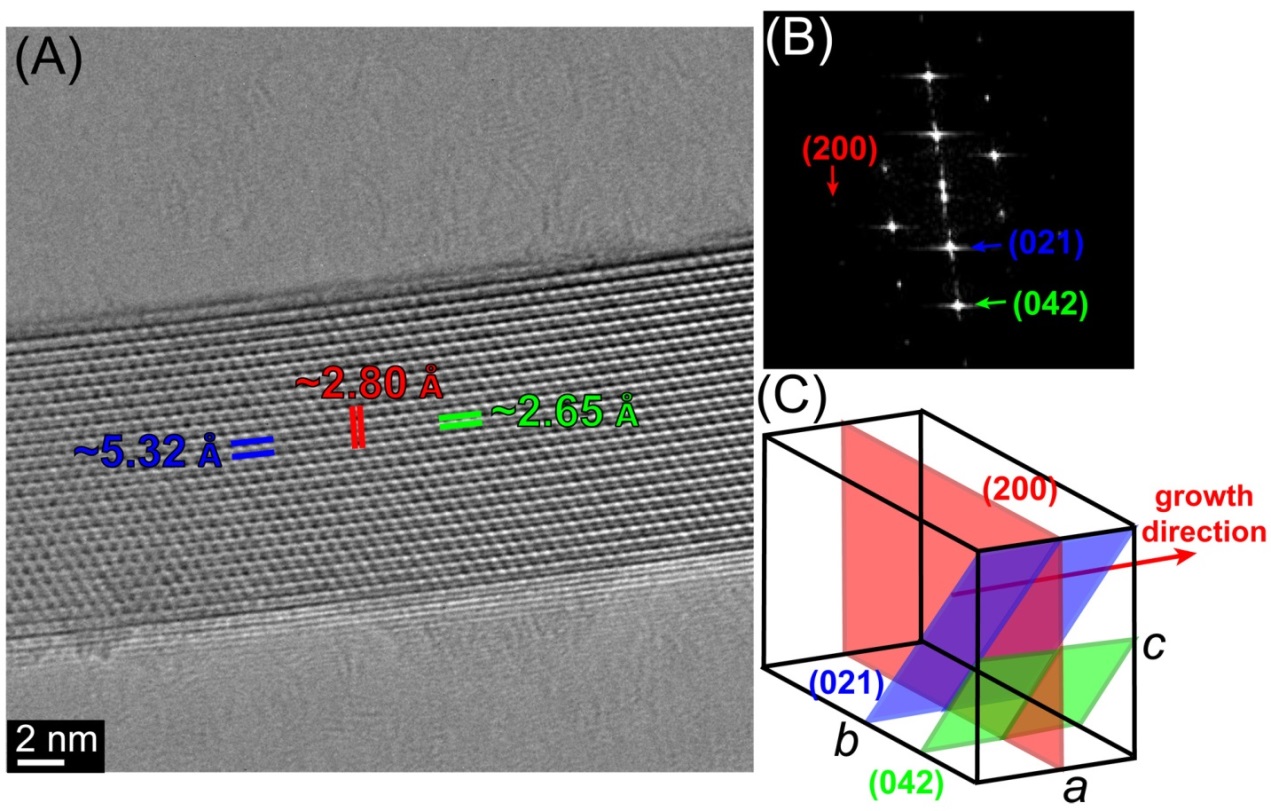


**Figure S5. FFT analysis.** **(A)** High resolution image of a NW. The crystalline structure is clearly discernible. The estimated values of the d-spacings between 3 sets of parallel planes are displayed and color-coded. **(B)** After comparing the d-spacing values and knowing that the set of green planes is parallel to the blue planes and both of these are orthogonal to the red planes (which extend along the growth direction) one can properly index the FFT pattern. Here, the correct set of Miller indexes obtained from the 34-1039 card which corresponds to the Al_5_BO_9_ phase. **(C)** A schematic drawing of the selected plane positions within the unit cell. One can readily observe how in effect the green and blue planes correspond to the parallel (042) and (021) planes respectively while the red planes are orthogonal to them both and correspond to the (200) indexes. The growth direction is along the red planes which lie parallel to the [100] direction which is in turn parallel to the octahedral chains that form the backbone of the Al_5_BO_9_ crystal structure.

Both phases of aluminium borate are built upon a backbone of parallel octahedral chains, however, they have different orientations with respect to the unit cell axis in each of the phases (see figure S6).


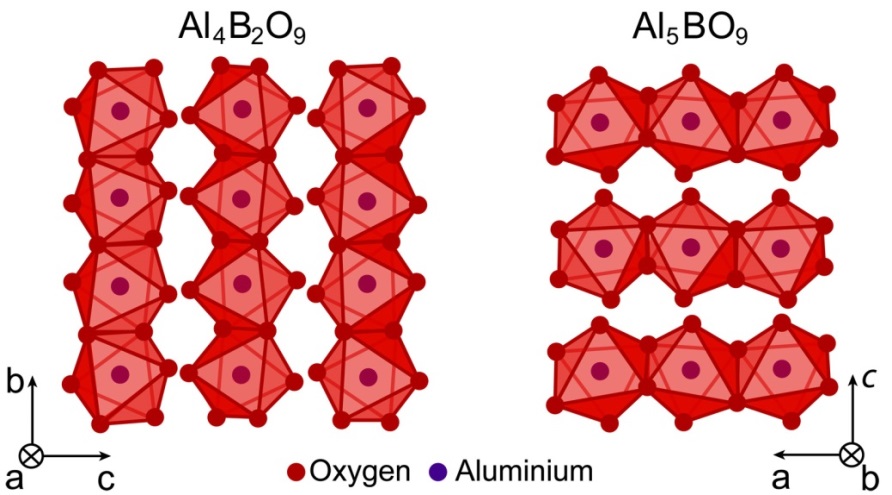


**Figure S6. Octahedral Backbones.** The backbone of the Al_4_B_2_O_9_ phase (left) constitutes linear chains made of octahedral AlO_6_ groups tightly packed together. The chains run parallel to the b axis of the unit cell. Whereas in the case of the Al_5_BO_9_ phase (right) identical chains running along the direction of the a axis of the unit cell. The interlinking AlO_X_ and BO_X_ groups have been omitted for clarity.


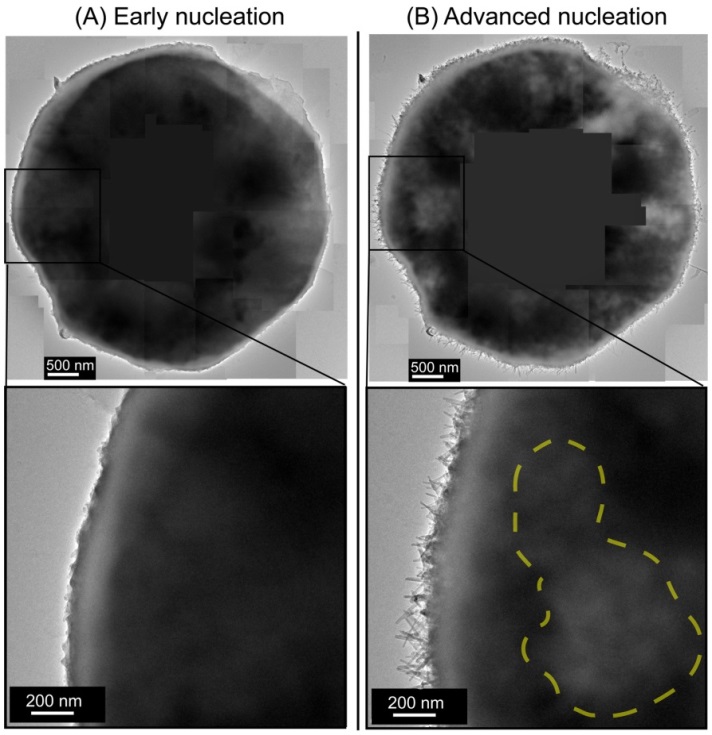


**Figure S7. Free volume creation.** **(A)** The precursor gets a slightly compacted as it first reacts to the incoming (broad) electron beam. **(B)** More patches of light contrast appear in several regions as soon as the beam waist is condensed after the initial quasi-instantaneous formation of NWs using broad beam irradiation. The use of a condensed beam is used to resume growth of the NWs, which we term extended growth. The light contrast patches reflect the removal of material due to beam-precursor interactions, viz., the creation of free volume within the precursor.

**Charging of TEM specimens.**

Suppose having a TEM specimen traversed by an electron beam. The charge balance equation of the irradiated volume can be expressed by:^1^

$\frac{dQ}{dt}=-I_{0} +I_{T} +I_{E} -I_{S} -I_{+}$ (1)

Where *dQ/dt* is the change rate of total charge *Q* contained within the irradiated volume, *I_0_* is the electric current of the main electron beam, *I_T_* is the transmitted beam, *I_E_* are the electrons emitted from the beam-film interactions, *I_S_*
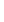
are the electrons from the regions of the specimen surrounding the illuminated volume that might flow into it and *I_+_* accounts for the possibility of having cations leaking outwards from the interaction volume. We know take a closer look at each one of the terms appearing in equation (1) to obtain a more detailed expression for the charge accumulation rate *dQ/dt*.

Good TEM specimens are sufficiently thin so as to allow the full beam to get transmitted through it, in this scenario one can approximate *I_T_ ≈ I_0_*. In somewhat thicker TEM specimens (some hundreds of nanometers) a fraction of the primary electrons can get trapped at a certain depth within the specimen. In general the transmitted current *I_T_* can be expressed as a fraction *mI*_0_ of the primary beam, where 0 ≤ m ≤ 1.

*I_S_* depends on the electrical properties of the irradiated specimen (its conductivity or alternatively, the amount of loose electrons occupying states on the conduction band of the material). If the electron irradiation can significantly change the composition of the material, such as for example by electron induced oxygen desorption through the Knotek-Feibelman (K-F) pathway,^2^ then *I*_S_(*t*) becomes a function of the irradiation time *t.* The specimen’s electrons making up the current *I*_S_(*t*) move into the irradiated volume through the surface *S* of the “walls” defined by the paths of the electrons as they traverse the specimen. If the irradiated volume can be approximated by a cylinder, then *S* corresponds to the area of the cylinder’s walls removing its caps. Thus, *I*_S_(*t*) it can be expressed as:

$I_{S}(t)=S\gamma(t)\left|\overrightarrow{E}(Q(t))\right|$ (2)

Where *γ* is the conductivity of the specimen which might also evolve over time and $\overrightarrow{E}$ is the electric field generated by the charge *Q*.

The term *I_E_* encompasses electrons being “ripped off” from the specimen’s nuclei via a variety of processes triggered by the main electron beam. The main contributions to *I_E_* are those of secondary electrons (SE) *I_SE_* and Auger electrons *I_AES_*. We deal first with aspects related to the production of SE.

The secondary electron yield *δ_SE_* (defined as the ration between the current due to SE and the main beam current *I_SE_ /I*_0_) can be a complicated function depending on the electrical properties of the specimen, the energy of the primary e-beam and the depth in the specimen at which the secondaries are generated. Secondary electrons have energies that fall below the 50 eV and the majority of them have energies under 10 eV.^1, 3, 4^ The secondary electron yield of all analyzed materials follows a universal trend. The yield *δ_SE_* rises from zero at a primary energy *E_0_* = 0 eV up to a maximum at a certain incident energy $E_{0}^{m}$(which typically is around 1 keV) and then falls monotonically as the at about 1/ *E_0_* as the energy rises^5^ (see Figure 1). This shared trend has justified the search of a so-called universal secondary electron yield curve^6, 7^ and this kind of approach is sufficiently satisfactory for our purposes.

The yield *δ_SE_* can be calculated from:

$\delta_{SE}(E_{0})=\int_{0}^{z} n(z,E)p(z)dz$ (3)

Where *n*(*z, E*) is the generation rate of secondary electrons at a depth *z* and *p*(*z*) is the probability of a given secondary to successfully leave a specimen and escape into the vacuum. *n*(*z,E*) is accurately given by Bethe’s expression:

$$n(z,E)=-\frac{1}{B_{SE}}\frac{dE}{ds}$$

Where *B*_SE_ is the average excitation energy to produce a secondary electron and *s* describes the trajectory of the primary electron through the specimen. The factor *dE/ds* refers to the stopping power of the specimen, i.e. the rate at which the primary electrons lose energy as they traverse the specimen. Assuming a simple dependency of the stopping power on the energy and range *R* of the primary electrons along the specimen:^5^

$-\frac{dE}{ds}=\frac{E_{0}}{R}\rho$ → $n(z,E)=\frac{1}{B_{SE}}\frac{E_{0}}{R}\rho$

Where *R* is the electron range in kg/m^2^ and $\rho$is the density of the traversed medium.

The probability *p*(*z*) assuming that the secondaries scatter symmetrically within the specimen can by written as:

$$p(z)=\frac{1}{2}e^{-\frac{z}{\lambda_{SE}}}$$

Where *λ*_SE_ is the effective scape depth of the secondaries. Computing the yield *δ_SE_* from equation (3) we get:

$$\delta_{SE}=\frac{1}{2}\frac{E_{0}}{B_{SE}}\frac{\lambda_{SE}}{R}\rho\left(1-e^{-\frac{R}{\lambda_{SE}}}\right)$$

Which is in line with previous derivations.^8, 9^ The magnitude of *I_SE_* can be directly estimated as:

$I_{SE}=\frac{I_{0}}{2}\frac{E_{0}}{B_{SE}}\frac{\lambda_{SE}}{R}\rho\left(1-e^{-\frac{R}{\lambda_{SE}}}\right)$ (4)

Now we turn our attention to the Auger current *I*_AES_. The Auger current *I*_AES_ (*z*,*E*_0_) generated at a certain depth z of a specimen bombarded by a primary beam of energy *E*_0_ can be approximated by:^1^

$I_{AES}(z,E_{0})\approx I_{0}\sum_{i} N_{Ai}\sigma_{i}(A_{i},E_{0})a_{Klm}^{i}\alpha_{Klm}^{i}z$ (5)

The sum runs over all the atomic species *A_i_* present the specimen. *N_Ai_* is the atomic density of the *A_i_* species in cm^-3^, $\sigma_{i}(A_{i},E_{0})$ is the interaction cross section of the *A_i_* species at incident energy *E*_0_ . $a_{Klm}^{i}$ measures the Auger yield involving emissions initiated at the core K-shell level of the species *A_i_* and leaving two holes in the *l-th* and *m-th* levels. The factors $\alpha_{Klm}^{i}$have been introduced in order to count only those Auger electrons that are emitted in the direction of the interface specimen-vacuum assuming that the Augers are not ejected along any preferential direction. The added factor $\alpha_{Klm}^{i}$ are defined as the ratio between the illuminated specimen-vacuum surface *a* (with a radius$r_{0}$ equal to the e-beam probe’s radius) and the area *A_Klm_* of the walls of the cylindrical volume within which the Auger electrons are produced. The height of the cylinder is defined by the effective escape depth $\Lambda_{Klm}^{i}$of a given Auger electron with an initial kinetic energy $E_{Klm}^{i}$. Thus we have that the factors $\alpha_{Klm}^{i}$are given by:

$$\alpha_{Klm}^{i}=\frac{a}{A_{Klm}}=\frac{\pi r_{0}^{2}}{2\pi r_{0}\Lambda_{Klm}^{i}+2\pi r_{0}^{2}}=\frac{r_{0}}{2(\Lambda_{Klm}^{i}+r_{0})}$$

A consequence of this reasoning is that there is only a discrete series of depths *z_i_* that are relevant to take into account. Those *z_i_* coincide with the effective escape depths $\Lambda_{Klm}^{i}$ since only Auger electrons generated within these discrete distances from the specimen’s surface are able to retain enough kinetic energy in order to escape it. Therefore, *I*_AES_ as given in equation (5) can be rewritten as:

$I_{AES}(z,E_{0})\approx I_{0}\sum_{i} N_{Ai}\sigma_{i}(A_{i},E_{0})a_{Klm}^{i}\alpha_{Klm}^{i}\Lambda_{Klm}^{i}$ (6)

We can write the Bethe’s the interaction cross section $\sigma_{i}(A_{i},E_{0})$in cm^-2^ with the notation we have employed:^10^

$$\sigma(A_{j},E_{0})=\frac{6.51\times10^{-14}}{E_{0}E_{K}^{i}}Z_{K}^{i}b_{K}^{i}\ln\left(\frac{c_{K}^{i}E_{0}}{E_{K}^{i}}\right)cm^{2}$$

Where $E_{K}^{i}$ is the binding energy of the K-shell if the species *A_i_*, $Z_{K}^{i}$ is the number of electrons in that shell, $b_{K}^{i}$ and $c_{K}^{i}$ are constants to be determined. Substituting into equation (6) we get a final expression to estimate the current made of Auger electrons that leave the specimen:

$I_{AES}\approx I_{0}\sum_{i} N_{Ai}\frac{6.51\times10^{-14}}{E_{0}E_{K}^{i}}\ln\left(\frac{c_{K}^{i}E_{0}}{E_{K}^{i}}\right)Z_{K}^{i}b_{K}^{i}a_{Klm}^{i}\alpha_{Klm}^{i}\Lambda_{Klm}^{i}$ (7)

Substituting *I_T_* = *mI*_0_, *I*_S_ from equation (2), *I*_SE_ from equation (4) and *I*_AES_ from equation (7) into equation (1) we finally get an expression to calculate the charge accumulation rate equation of an irradiated specimen.

$\begin{matrix} \frac{dQ(t)}{dt}\approx I_{0}\left[-1+m+\frac{E_{0}\lambda_{SE}\rho}{2RB_{SE}}\left(1-e^{-\frac{R}{\lambda_{SE}}}\right)+\sum_{i} N_{Ai}\frac{6.51\times10^{-14}}{E_{0}E_{K}^{i}}\ln\left(\frac{c_{K}^{i}E_{0}}{E_{K}^{i}}\right)Z_{K}^{i}b_{K}^{i}a_{Klm}^{i}\alpha_{Klm}^{i}\Lambda_{Klm}^{i}\right] \\ -S\gamma\left|\overrightarrow{E}(Q)\right|-I_{+} \end{matrix}$…(8)

Some of the parameters in equation (8) are in control of the experimenter such as *I*_0_, the beam waist radius and *E*_0_ while the remaining ones are determined by the composition and geometry of the specimen.

It is important to notice that equation (8) cannot be solved analytically since there are several factors that are dynamically locked in “feedback loops”. For instance, as charge accumulates in the irradiation volume an electric field starts to build up. The field is a byproduct of the SE and Auger leakages but the field potential itself directly damps the probability of the SE and Auger electrons to successfully leave the specimen. The *I*_SE_ and *I*_AES_ currents play a central role on the charge accumulation rate and conversely the accumulated charge (through the generated electric field) damps the intensity of these same currents. This type of feedback loops call for a numerical approach (simulation) to solve equation (8). Nevertheless, in what follows we will take the case of a specimen made of B_2_O_3_ and make some judicious idealizations in order to get some insights on how the charge accumulates within a small irradiated volume.

**Charge accumulation and dielectric breakdown of a B_2_O_3_ specimen irradiated by a condensed electron beam.**

Our specimens can be idealized as made of amorphous B_2_O_3_ since their nominal constitution is of around 60 % oxygen, 36 % boron and 4 % of aluminum and a significant fraction of that 4% Al gets readily used in forming the early nucleated NWs. Our specimens are certainly thicker than ideal TEM specimens. Readings of the electron beam current hitting on the fluorescent screen when the beam path is unimpeded and when the condensed beam travels through the specimen determined that around 90 % of the main beam gets transmitted through. Thus we should in principle take *I_T_* ≈ 0.9 *I*_0_. However, is difficult to determine if most the missing electrons are in fact absorbed by the specimen or strongly scattered at high deflection angles. Furthermore, we are more interested in looking at the charge accumulated near the specimen’s surface, more specifically, within the volume from where the SE and Auger electrons are emitted and can successfully leave the specimen. This region has a depth slightly larger than 10 nm (as we will see in brief) and it is safe to consider than *I_T_* ≈ *I*_0_ through this superficial section of the specimen so these terms cancel each other out in equation (8). The electric field produced by absorbed electrons much deeper in the specimen will be ignored for the rest of this discussion since it is generated by a much more diffuse charge density.

Let us first look at the *I*_SE_ contribution before dealing with that of the Auger electrons and the inflowing electrons *I*_S_. Despite the fact that secondaries have low kinetic energies, insulating materials have relatively higher effective escape depths since their wide band gap prevents the low energy SE from promoting valence band electrons into the conduction band, instead, the secondaries lose energy mainly through electron-phonon interactions.^11, 12^ The effective length of secondaries arising from the cascades initiated by Auger emissions initiated by the excitation of K shell electrons with binding energies in the range of 200 eV (such as the boron K-edge at 193.4 eV)^10^ as determined by Total Electron Yield (TEY) measurements is of around λ_SE_ ≈ 6 nm.^13^ The average excitation energy *B*_SE_ for boron oxide is of 99.6 eV and the range R of 300 kV electrons through a B_2_O_3_ specimen is of around 9.69 g/cm^2^.^15^

Taking the density of B_2_O_3_ as $\rho$=2.460 g/cm^3^ we then have all the parameters needed to determine the magnitude of *I*_SE_ as given by equation (4).

We now turn our attention to the Auger contribution. The Auger spectrum of B_2_O_3_ has been measured.^15, 16^ The spectrum shows three main peaks at around 143 eV (lowest intensity), 158.5 eV (middle intensity) and 169 eV (highest intensity). According to Rogers and Knotek all the Auger emissions start at an excitation of one 2 core-level electrons (*Z*_K_ = 2) of the boron K-shell located at *E*_K_ = -193.4 eV with respect to the Fermi energy. The Auger emission at 143 eV leaves two holes in the O(2s) level at -26.3 eV after filling up the initial vacancy in the B(1s) level, changing the initial O^2-^ anion into a O^0^ atom and the initial B^3+^ cation remains unchanged. The peak at 158.5 eV leaves a hole in a O(2p) level at -7.2 eV and one in the O(2s) level at -26.4 eV from which the Auger electron is emitted. The process leaves a O^0^ and a B^3+^ ions behind. Finally, the Augers at 169 eV leave a hole in the B(2p) level at -11.2 eV and another in a O(2p) level at -11.5 eV from where the Auger electron is emitted. A O^-^ anion and a B^4+^ cation are left in the specimen.

To set everything in accord with the adopted nomenclature *E_Klm_* we have *E_Kss_* = 143 eV, *E_Kps_* = 158.5 eV and *E_Kpp_* = 169 eV and each one of these emissions has an associated effective escape depth Λ*_Kss_*, Λ*_Kps_* and Λ*_Kpp_*. It is important to notice that the superindex *i* can now be dropped since all the Auger emissions are initiated at the K-shell level of B, i.e. no need to sum over distinct atomic species *A_i_* in equation (7).

The effective escape length depends on the inelastic mean free paths *λ_Klm_* of the corresponding electron. Sjazman et al. obtained an approximation for calculating *λ_Klm_*^18^ of an electron with kinetic energy in the range of 50 – 100 eV:

$\lambda_{Klm}\mathop{(A}\limits^{\circ} )\approx1.8\overline{E}\frac{E_{k}^{3/4}}{E_{p}^{2}}$ (9)

Where *E_p_* refers to the plasmon energy that, according to the studies performed by Joyner and Hercules,^16^ is of around $E_{p}$≈ 10.8 ±0.5 eV for boron oxide. $\overline{E}$is the centroid of the energy loss function of the travelling electron that for the case of insulators can be approximated by $\overline{E}\cong E_{p}+E_{g}$ where $E_{g}$is the bang gap energy^18^ which is around 7 eV for B_2_O_3_, then,$\overline{E}_{B_{2}O_{3}}\cong17.8$eV. In order to calculate the effective escape depth for a given Auger electron Λ*_Klm_* we need to determine the number of inelastic mean free paths *λ_Klm_* that the electron can travel before its kinetic energy falls below 50 eV (since the approximation in equation (9) becomes non-valid for kinetic energies ˂50 eV, i.e. the typical energy of SE). After this threshold the electrons are assumed to be able to travel 6 nm further through the specimen since this is the effective escape value for SE experimentally measured for B_2_O_3._^13^ The calculation has to be done in an iterative manner since the IMPF *λ_Klm_* changes as a function of energy and this quantity diminishes by $\overline{E}$ from one collision to the next, thus, the IMPF $\lambda_{Klm}^{1}$ and $\lambda_{Klm}^{2}$ separated by one collision will be different from one another. A simple algorithm can be implemented to calculate Λ*_Klm_* as the sum of *n* inelastic mean free paths $\lambda_{Klm}^{n}$ where the index *n* stops running as soon as the electron’s kinetic energy *E_Klm_* of falls below 50 eV plus the effective escape depth λ_SE_:

$$\Lambda_{Klm}=1.8\overline{E}\sum_{n=0} \frac{\left(E_{Klm}^{n}-n\overline{E}\right)^{3/4}}{E_{p}^{2}}+\lambda_{SE}$$

Where $E_{Klm}^{0}$ is the initial kinetic energy of the original Auger electron. Using this method we can compute the effective escape depths Λ*_Klm_* for all the Auger emissions of B_2_O_3_. The results are shown in Table 1.

| **Material** | **Initial kinetic energy of the Auger emission *Klm*** | **Effective escape depth of the Auger emission *Klm*** |
| --- | --- | --- |
| B_2_O_3_ | *E_Kss_* = 143 eV  *E_Kps_* = 158.5 eV  *E_Kpp_* = 169 eV | Λ*_Kss_* = 11.5 nm  Λ*_Kps_* = 12.6 nm  Λ*_Kpp_* = 13.2 nm |

**Table 1. Effective escape depths for the Auger electrons of B_2_O_3_.**

Since the Auger yield for elements between Li and Ar is nearly equal to unity^1^ we can assume that the sum of the $a_{Klm}$ factors is equal to 1. We can look at the relative heights of the Auger peaks of the spectrum reported by Rogers and Knotek in order to determine the corresponding fractions that assigned to each $a_{Klm}$.^15^ Doing this we get $a_{Kss}$ ≈0.23, $a_{Kps}$ ≈ 0.37 and $a_{Kpp}$≈ 0.40.

If we consider that the specimen is irradiated by an electron probe with a diameter of 100 nm, then, the factors $\alpha_{Klm}$for the Auger electrons of B_2_O_3_ we have: $\alpha_{Kss}$= 0.41, $\alpha_{Kps}$= 0.40 and $\alpha_{Kpp}$= 0.39. All values are close to 0.5, which means that the escaping electrons come from a volume that resembles a thin disk rather than a tall cylinder.

Unfortunately we could not find approximations for the factors $b_{K}$ and $c_{K}$ that are relevant for calculating the interaction cross section of the K-shell of boron, however, Powell makes a recollection of the values of both coefficients for Be and C which are the immediate neighbors of B in the periodic table.^10^ One could interpolate from the coefficients of Be and C to obtain $b_{K}$ and $c_{K}$ for B after noticing that $b_{K}$ does not vary for more than 0.10 and $c_{K}$ for more than 0.13 between Be and Ne (according to the values reported by McGuire). Doing this we obtain $b_{K}$ = 0.88 and $c_{K}$= 0.83 for B.

Calculating the density of B atoms *N_B_* =4.26 x 10^28^ atoms/cm^3^ we just get all the factors needed to compute the *I*_AES_ contribution as displayed in equation (7).

Concerning the contribution *I*_S_ we need to take into account that B_2_O_3_ is a good insulator (band gap ~ 7 eV),^15, 16^ thus, its poor electrical conductivity grants that initially one can take *I_S_* ≈ 0 A. However, it has been proven that an electron beam can induce reduction reactions on superficial boron oxide islands subjected.^15^ Gradually, the electrical conductivity of the specimen increases as the relative content of pure elemental B (band gap ~1.4 - 1.5 eV)^16^ grows within the irradiated region, i.e. *I_S_*(*t*) becomes an increasing function of the irradiation *t* time and it can no longer be safely neglected.

The factor I_+_ in equation (8) can be neglected on grounds of the low relatively low mobility of positively charged ions in comparison with much lighter electrons. Initially, at time *t* = 0 s, the specimen is to be considered as entirely made of B_2_O_3_, thus, *I*_S_ can be regarded as negligible. With all of these considerations in place, equation (8) can be tuned for the case of a B_2_O_3_ yielding the following expression:

$\begin{matrix} \frac{dQ(t)}{dt}\approx I_{0}\left\{\frac{\alpha_{SE}E_{0}\lambda_{SE}\rho}{2B_{SE}R}\left(1-e^{-\frac{R}{\lambda_{SE}}}\right)+\frac{1.14\times10^{-13}N_{B}}{E_{K}E_{0}}\ln\left(\frac{0.83E_{0}}{E_{K}}\right)\left[\alpha_{Kss}a_{Kss}\Lambda_{Kss}+\alpha_{Kps}a_{Kps}\Lambda_{Kps}+\alpha_{Kpp}a_{Kpp}\Lambda_{Kpp}\right]\right\} \\ \end{matrix}$…(9)

The factor 1.14 x 10^-13^ arises from multiplying the 6.51 x 10^-14^ by the factor *b_j_* = 0.88.

After a certain time *t*_1_ a significant amount of oxygen will be desorbed from the irradiated volume (mainly through the Auger emission cascades as described by the K-F Feibelman mechanism) effectively transforming a portion of the initial B_2_O_3_ into elemental B. Since B has a larger conductivity than B_2_O_3_, after *t*_1_ the contribution of inflowing electrons *I*_S_ to the irradiated volume cannot be safely neglected anymore. On a first approximation we can estimate the desorption rate of oxygen atoms by realizing that the byproducts of the Auger transitions at *E_sss_* =143 eV and *E_sps_* = 158.5 eV of B_2_O_3_ are neutral O^0^ atoms that get easily desorbed. Thus, we can calculate the amount of desorbed atoms after a second of irradiation as follows:

$\frac{dO^{0}}{dt}=\frac{1}{2e}I_{0}\frac{1.14\times10^{-13}N_{B}}{E_{K}E_{0}}\ln\left(\frac{0.83E_{0}}{E_{K}}\right)\left[\alpha_{Kss}a_{Kss}\Lambda_{Kss}+\alpha_{Kps}a_{Kps}\Lambda_{Kps}\right]$ (10)

Where *e* is the charge of the electron in Coulombs and the factor ½ accounts for the fact that there is a single O^0^ atom produced for every 2 Auger electrons.

If we irradiate the B_2_O_3_ specimen with a 300 kV beam having a total current of *I*_0_ = 20 nA squeezed into a probe with a radius $r_{0}$= 50 and take the height of the irradiated disk of interest as equal to $\Lambda_{Kpp}$ (the longest effective escape length of the Auger emissions), then the number of oxygen atoms desorbed in an irradiation period of 1 s can be estimated from equation (10). This renders an oxygen desorption rate of around 1.10 x 10^5^ O atoms/s, this amounts to less than 2% of the B_2_O_3_ contained within the irradiated disk turned into elemental B per second. This simplified result grants that during this first second of irradiation the specimen can be considered as made of almost entirely B_2_O_3_ implying that the *I_S_* contribution can still be safely neglected. Only after almost 60 s of continued irradiation the whole B_2_O_3_ contained inside the superficial irradiated disk would be turned to elemental B at this desorption rate.

Now we can calculate the charge accumulation rate of a disk with height $\Lambda_{Kpp}$= 13.2 nm irradiated for 1 second by a probe with radius $r_{0}$= 50 nm using equation (9). We get that the charge *Q* accumulated inside the irradiated disk is of around 3.51 x 10^-12^ C (which is equivalent to around 22 millions of electrons escaping the irradiated volume).

The net accumulated charge produces an electrostatic potential whose radial component *F_R_*(r) extending over the surface of the irradiated specimen. The maximum of *F_R_*(*r*) occurs at the perimeter of the beam profile, i.e. at *r* =$r_{0}$ and can be estimated by:^19^

$F_{R}(r_{0})\cong\frac{\rho_{C}z}{\varepsilon}$ (11)

Where $\rho_{C}$is the charge density inside the irradiated disk with height *z* = $\Lambda_{Kpp}$ and ε is the dielectric constant of the specimen. $\rho_{C}$after 1 s of irradiation is simply $\rho_{C}$=3.51 x 10^-12^ C /π$\Lambda_{sps}$ $r_{0}$^2^ and the dielectric constant of B_2_O_3_ can be approximated by that of borosiliciate glass ε ≈ 3.54 x 10^-11^F/m (since we could not find the value for ε of B_2_O_3_ in the literature). Inserting these values into equation (11) we get *F_R_* ≈ 1.26 x 10^13^ V/m which is much higher than the dielectric field strength of most insulators (~10^9^ V/m for silica). Even if one leaves aside the contribution of *I*_SE_ on the grounds of SE having low kinetic energies (so they are rapidly reabsorbed into the specimen due to the increasingly high positive potential) we still get a total accumulated charge of *Q* ≈ 6.05 x 10^-14^ C after 1 second of irradiation. The radial component of the electrostatic potential associated with this charge is of around 2.17 x 10^11^ V/m that is still higher than the dielectric field strength of most insulators.

In both estimations (with and without *I*_SE_) the irradiated region of the specimen undergoes electrostatic breakdown at the borders of the irradiated disk and is forced to emit an avalanche a positively charged ions in order to recover electrostatic equilibrium. It is hypothesized that these ions correspond mainly to positively charged boron and oxygen species which make the bulk of the feedstock material used in the growth of the NWs. The Al ions/atoms (which are much fewer) might be transported along the avalanche of B and O ions, or, might be previously deposited over the lacey C support at the moment of initial nucleation when a substantial amount of the precursor material spreads over the underlying C film of the TEM grid.

**Some potentially relevant aspects that had been put aside.**

Some of the considerations that have been put aside for the estimation of the charge accumulation rate that has been just performed are the following:

- The minimum kinetic energy that a secondary electron must have in order to escape from the surface of an insulator is equal to the electron affinity of the material *χ* which is defined as the difference between the vacuum level and the conduction band minimum. In most insulators the electron affinity is of the order of 1 eV.^12^ This has been ignored for the time being but it would only reflect on a marginally shorter height of the disk out of which secondary electrons can escape, i.e. a smaller volume inside which the charge *Q* is being accumulated.
- The electrostatic potential created by trapped primary electrons deeper within the specimen. A B_2_O_3_ specimen with a thickness of around 600 nm would be needed to stop the majority of the incoming electrons if one takes into account their large ranges.^14^ Our measurements of the transmitted current tell us that our specimens are significantly thinner since the majority of the incoming electrons manage to traverse them completely. However, it would be interesting to perform a Monte Carlo simulation to estimate the fraction of trapped electrons and the potential field associated with it. The results could determine the influence of the associated field on the positively charged ions expelled after the specimen undergoes electrostatic breakdown.

**Generation differences.**

We have portrayed a general scheme for the generation and transport of feedstock material, however, there might be subtle but important differences in the production mechanisms for each kind of ion. Now we address some of the most relevant issues that pinpoint some of the different aspects that characterize the production of Al, B and O.

Oxygen, boron and aluminium ions are produced in different proportions. We postulate that the Auger events that outline the K-F mechanism are the main responsible process feeding ions into the leak current *I_+_*. In an “ideal” situation an Auger cascade will produce a free O^+^ (or O^0^) and for each oxygen ion being released there will be a metallic (B or Al) cation left behind. Whether the cation is free (or not) depends on complicated structural details of the specimen’s matrix, thus, it is not straightforward to know if this cation will feel a repulsive force strong enough as to integrate it into *I_+_*. Furthermore, there is not necessarily a one-to-one correspondence between the production of a O^+^ and a metallic M^+^ ion, even the production of a neutral oxygen leaves a metallic ion behind. Thus, we can only postulate that it is more likely to produce B ions by this sort of process simply because there is much more B in the specimen than Al (36% of the former against 4% of the latter).

All in all, it is likely that the generation profile of each ionic species is distinct and that it follows a very characteristic time/concentration evolution. All the ions migrate along surfaces however; their relative proportions might vary over time.

**Temperature increase in the precursor due to beam heating.**

Finally, to confirm the athermal nature of the phase transition in that the formation of NWs occurs with a negligible temperature rise caused by the electron beam impinging upon the precursor material.

We need to check if the beam heating effects have any important role to play during the NW formation. This can be done easily under the assumption that the precursor can be viewed as a thin B_2_O_3_ film (remember that only 4 % of its atoms are Al).

The point of maximum temperature in the irradiated precursor is located at the center of the beam (where the origin is conveniently set). According to Liu et al.^20^ the temperature increase at the beam’s center once the steady heat flow state is attained is given by:

(12)

Where, *W*_0_ is the total absorbed power, *z* is the precursor’s thickness, *κ* is the thermal conductivity of the film, *R_S_* is the radius of the specimen and *r_0_* is the beam radius. *W*_0_ can be calculated from the expression:

Where *Q* is the total linear energy loss of 300 kV electrons travelling through B_2_O_3_ which can be extracted from the tables of Pages et al.^14^ *I*_0_ is the beam current and
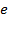
 the electron charge.

Then using equation (12), for a beam current of 20 nA, a beam radius of 60 nm, a specimen 2 microns across (current density 177 A/cm^2^), we obtain a temperature rise of
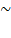
37 K above room temperature at the center of the beam. This temperature informs us that the role of heating by the electron beam in the quasi instantaneous formation of aluminium borate nanowires is negligible when using a broad irradiating electron beam given that the melting point of B_2_O_3_ is of around 450 °C.

**Video Legend**

In situ observations of Aluminum Borate Nanowire growth.

**References.**

1. Cazaux, J. Correlations between ionization radiation damage and charging. *Ultramicroscopy.* **60,** 41 l–425 (1995).
2. Knotek, M. L. & Feibelman, P. J. Stability of ionically bonded surfaces in ionizing environments. *Surf. Sci.* **90,** 78 – 90 (1979).
3. J. Cazaux. The role of the Auger mechanism in the radiation damage of insulators. *Microsc. Microanal. Microstruct.* **6,** 345 – 362 (1995).
4. F. J. Pijper, P. Kruit, Detection of energy-selected secondary electrons in coincidence with energy-lass events in thin carbon foils, *Phys. Rev. B.* **44,** 9192 – 9200 (1991).
5. Y. Lin, D. C. Joy, A new examination of secondary electron yield data, *Surf. Interface Anal.* 37, 895 – 900 (2005).
6. H. Seiler, Secondary electron emission in the scanning electron microscope. *J. Appl. Phys.***54,** R1 (1983).
7. A. J. Dekker, in: Solid State Physics, Advances in Research and Applications, eds. F. Seitz and D. Turnbull (Academic Press, New York, 1958) p. 251.
8. G. F. Dionne, Effects of secondary electron scattering on secondary emission yield curves, *J. Appl. Phys.* **44,** 5361 (1973).
9. J. J. Scholtz, D. Dijkkamp, R. W. A. Schmitz, Secondary electron emission properties, *Philips J. Res.* **50,** 375 – 389 (1996).
10. C. J. Powell, Cross sections for ionization of inner-shell electrons by electrons, *Rev. Mod. Phys.* **48,** 33 – 47 (1976).
11. R. C. Alig, S. Bloom, Secondary‐electron‐escape probabilities, *J. Appl. Phys.* **49,** 3476 (1978).
12. A. Shih, J. Yater, C. Hor, R. Abrams, Secondary electron emission studies, *Appl. Surf. Sci.* **111,** 251 – 258 (1997).
13. M. Kasrai, M. E. Fleet, S. Muthupari, D. Li, G. M. Bancroft, Surface modification study of borate materials from B K-edge X-ray absorption spectroscopy, *Phys. Chem. Minerals.* **25,** 268 – 272 (1998).
14. L. Pages, E. Bertel, H. Joffre, L. Sklavenitis, Energy loss, range, and bremsstrahlung yield for 10-keV to 100-MeV electrons in various elements and chemical compounds. *Atomic Data*. **4,** 1-127 (1972).
15. Rogers, J. W. & Knotek, M. L. The oxidation of polycrystalline boron: the interpretation of AES and ELS results. *Appl. Surf. Sci.* **13,** 352 – 364 (1982).
16. D. J. Joyner, D. M. Hercules, Chemical bonding and electronic structure of B_2_O_3_, H_3_BO_3_, and BN: An ESCA, Auger, SIMS, and SXS study, *J. Chem. Phys.* **72,** 1095 – 1107 (1980).
17. A. Jablonski, M. Krawczyk, B. Lesiak, Influence of the matrix on boron detection by auger electron spectroscopy (AES), *J. Electon. Spectrosc. Relat. Phenom.* **46,** 131 – 143 (1988).
18. J. Szajman, J. Liesegang, J. G. Jenkin, R. C. G. Leckey, Is there a universal mean-free-path curve for electron inelastic scattering in solids, *J. Electron. Spectrosc. Relat. Phenom. 23,* 97 – 102 (1981).
19. J. Cazaux, Some considerations on the electric field induced in insulators by electron bombardment, *J. Appl. Phys.* **59,** 1418(1986).
20. M. Liu, L. Xu, X. Lin, *Scanning.* **16,** 1-5 (1994).
